# Evaluating Behavior Recognition Pipeline of Laying Hens Using Wearable Inertial Sensors

**DOI:** 10.3390/s23115077

**Published:** 2023-05-25

**Authors:** Kaori Fujinami, Ryo Takuno, Itsufumi Sato, Tsuyoshi Shimmura

**Affiliations:** 1 Division of Advanced Information Technology and Computer Science, Institute of Engineering, Tokyo University of Agriculture and Technology, Tokyo 184-8588, Japan; 2 Department of Bio-Functions and Systems Science, Graduate School of Bio-Applications and Systems Engineering, Tokyo University of Agriculture and Technology, Tokyo 184-8588, Japan; 3 Department of Agriculture, Graduate School of Agriculture, Tokyo University of Agriculture and Technology, Tokyo 183-8509, Japan; 4 Institute of Global Innovation Research, Tokyo University of Agriculture and Technology, Tokyo 183-8509, Japan

**Keywords:** animal activity recognition, wearable sensors, inertial measurement units, animal welfare, machine learning

## Abstract

Recently, animal welfare has gained worldwide attention. The concept of animal welfare encompasses the physical and mental well-being of animals. Rearing layers in battery cages (conventional cages) may violate their instinctive behaviors and health, resulting in increased animal welfare concerns. Therefore, welfare-oriented rearing systems have been explored to improve their welfare while maintaining productivity. In this study, we explore a behavior recognition system using a wearable inertial sensor to improve the rearing system based on continuous monitoring and quantifying behaviors. Supervised machine learning recognizes a variety of 12 hen behaviors where various parameters in the processing pipeline are considered, including the classifier, sampling frequency, window length, data imbalance handling, and sensor modality. A reference configuration utilizes a multi-layer perceptron as a classifier; feature vectors are calculated from the accelerometer and angular velocity sensor in a 1.28 s window sampled at 100 Hz; the training data are unbalanced. In addition, the accompanying results would allow for a more intensive design of similar systems, estimation of the impact of specific constraints on parameters, and recognition of specific behaviors.

## 1. Introduction

Recently, animal welfare has been gaining interest worldwide; however, livestock management laws following animal welfare have been developed generally in European countries. Animal welfare refers to an animal’s physical and mental state concerning the conditions under which it lives and dies [1]. Particularly, five freedoms [2] should be considered basic concepts in animal welfare: (1) freedom from hunger or thirst; (2) freedom from discomfort; (3) freedom from pain, injury, or disease; (4) freedom to express (most) usual behavior; and (5) freedom from fear and distress. Laying hens have been reared in battery cages (conventional cages) consisting of several cage layers equipped with feeders, water suppliers, and egg outlets. Although this method is highly productive, the cage space per bird is small, thus restricting the expression of instinctive behaviors such as dust bathing and comfort behaviors, violating the third and fourth freedoms. Thus, rearing systems are among the most active topics in animal welfare research on layers, and attempts to develop welfare-oriented rearing systems while increasing productivity are underway. The Office International des Epizooties (OIE) (now World Organization for Animal Health) is developing a guideline, “Animal Welfare and Laying Hen Production Systems.” Hence, the presence or absence of behaviors such as dust bathing, fear, resting behaviors, physical condition, water and feed intake, illness incidence, mortality rate, and egg-production performance may be used to assess the welfare of layers [3]. Because these normal behaviors can be observed in cage-free systems such as floor rearing and multi-tiered aviary systems, where laying hens can move freely in a wide range of areas in a hen house, the dynamic transition from cage to cage-free has occurred.

The layers’ behaviors are identified visually at regular intervals (e.g., 3 min [4], 10 min [5], 60 min [6]) from video recordings or direct visual scans; however, this method requires considerable time for detailed observation and relies on the observer’s ability. Therefore, automatic observation methods have been explored and classified into two major categories: those using images recorded by cameras installed in the environment (i.e., image-based [7,8,9]), and those using sensors attached to individual hens (i.e., wearable sensor-based [10,11,12,13]). The image-based methods enable the observation of many hens simultaneously without any constraint on the individual hens. However, tracking each individual’s behavior is challenging in an environment where the structures are complex and occlusion is likely to occur, such as in an aviary of layers or where a number of hens are flying around. Moreover, the wearable sensor-based system requires hens to wear sensor units with unique IDs, which help in readily tracking their activity; an image-based approach cannot do this because occlusion may occur if there are numerous hens. Therefore, continuous monitoring of a hen’s behavior is possible, and a satisfactory understanding on the welfare of hens is expected. Thus, we take the wearable sensor-based approach and attach inertial sensors (i.e., accelerometers and angular velocity sensors) to layers. We believe that attaching sensors to all individuals in order to observe hundreds of hens in an actual poultry farm is impractical. In this case, we attach the sensors to a few sampled hens.

Wearable sensor-based behavior recognition has already been applied for conservation and management in wild animals [14,15,16] and healthcare and communication with humans for pets [17,18]. Wearable sensors have also been attached to livestock such as cows [19,20,21], sheep [13,22], and goats [23] for welfare and production management. However, minimal work exists in chicken behavior recognition using wearable sensors, and the target behaviors are limited to two to six types [10,11,12,13,24,25,26,27]. In contrast, we aim to increase comprehensiveness by extending the recognition target to 11 behaviors, thus enabling a multifaceted welfare evaluation. In addition, there has been little in-depth evaluation of the components of the chicken behavior classification system, such as sensor modality, sampling frequency, window length in time, beneficial sensor modalities, and classifier. The behavior recognition model is constructed using supervised machine learning. Unlike human behavior recognition, the training data for animal behavior recognition generally constitute a significant imbalance in the data amount among behavior classes because animals are not cooperative in data collection like humans [12]. Herein, we aim to understand handling imbalanced data by existing balancing methods. The findings of this study may provide useful information for designing similar systems by showing the impact of the components on the system’s performance, which would allow for improving the rearing system based on continuous monitoring and quantifying a large number of behaviors.

The remainder of this study is organized as follows: Section 2 presents the data collection, including the target behavior classes, labeling method, and the collected data statistics. The relevant components of the behavior recognition pipeline are defined in Section 3, which also presents the evaluation schemes. Section 4 demonstrates the in-depth experimental results listed above, followed by a discussion in Section 5. Finally, Section 6 concludes the article.

## 2. Data Collection

### 2.1. Study Site and Animals

The study was conducted for four days from April to July 2020 between 10:00 a.m. and 4:00 p.m., when the hens were considered to be more active, in indoor floor rearing of the agricultural department at Tokyo University of Agriculture and Technology, Tokyo, Japan. The Animal Experiment Committees of Tokyo University of Agriculture and Technology approved all animal experiments (Permit Number: R03-134). We used eight commercial layers (Boris Brown) at 61 weeks of age at the beginning of the experiment.

### 2.2. Method

**Figure 1:**
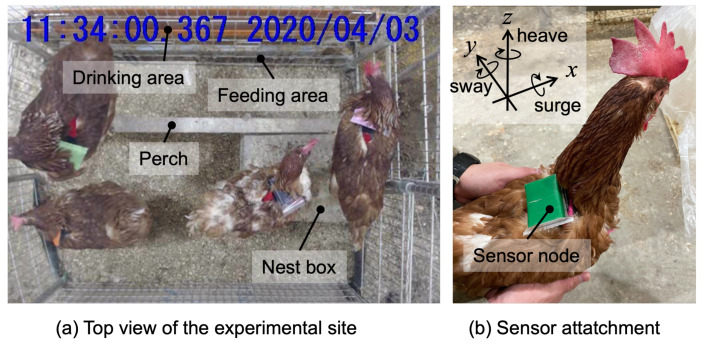
Experimental site and sensor attachment.

We caged four hens in a 100 cm × 76 cm floor rearing with a nest box, a perch, and a feeding and drinking area, as depicted in Figure 1. The floor was covered with wood shavings. The data for four hens were collected simultaneously. The reason for separating two groups of four hens is that four is an appropriate density to observe various behaviors.

We used a Bluetooth-based wireless inertial measurement unit (TSND151; ATR-Promotions Inc., Kyoto, Japan, weight 27 g) containing a three-axis accelerometer (±19.62 m/s${}^{2}$ (±2 G)) and three-axis angular velocity sensor (±500 degree/s) (i.e., gyroscope). We sampled the data at 1000 Hz, which were later down-sampled to verify the impact of sampling frequency on the recognition accuracy. We confirmed approximately 7.5 h of measurement at 1000 Hz. Each hen wore a harness designed for small dogs for a week before actual measurement for habituation, and the measurement unit was positioned in a dustproof and waterproof bag. It was attached to the harness to be on the hen’s back. Colored markers were attached to the surface of each sensor and used for individual identification during labeling. Additionally, colored makers were utilized for automatic position detection; however, this study does not consider automatic position detection.

Furthermore, for labeling by human annotators, the behaviors were video-recorded at 30 fps using a web camera (C920 HD Pro; Logicool Co., Ltd., Tokyo, Japan) positioned at the top of the cage to capture the entire coop. We collected data from wireless sensors and the video data on the same computer (CPU: Intel Core i7 7700 3.6 GHz, Intel, Santa Clara, CA, USA; Memory: 32 GB, OS: Windows 10, Microsoft Corporation, Redmond, WA, USA) running software (ALTIMA; ATR-Promotions Inc., Kyoto, Japan) for recording sensor data and video images synchronously.

### 2.3. Labeling

The collected accelerometer and angular velocity data were labeled by experienced human annotators, in which software (SyncPlay; ATR-Promotions Inc., Japan) was utilized to annotate the start and end periods of particular behaviors by observing the recorded video. We selected the target behaviors to enable a multifaceted welfare evaluation with categories including grooming, food seeking, migration, resting, food and water intake, and self-defense. Furthermore, various behaviors were observed during the data collection, such as pecking sensors, stretching, and breaking balance, which were included in the recognition targets. Because more ambiguous behaviors would appear in an actual environment, we considered it crucial to design a classifier under such an assumption and labeled it as one class (“Others”) that includes miscellaneous behaviors. In total, 12 behaviors, including “Others,” were labeled as depicted in Table 1.

**Figure 2:**
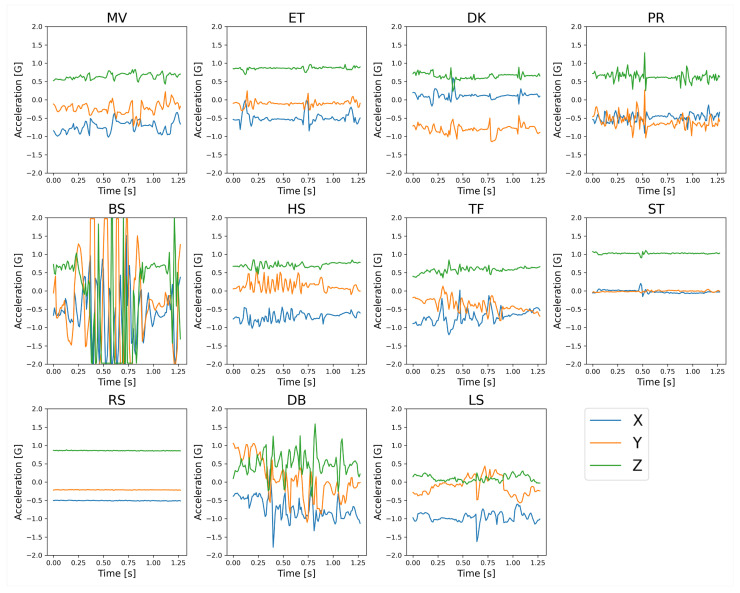
Examples of acceleration signals of each behavior, down-sampled to 100 Hz.

**Figure 3:**
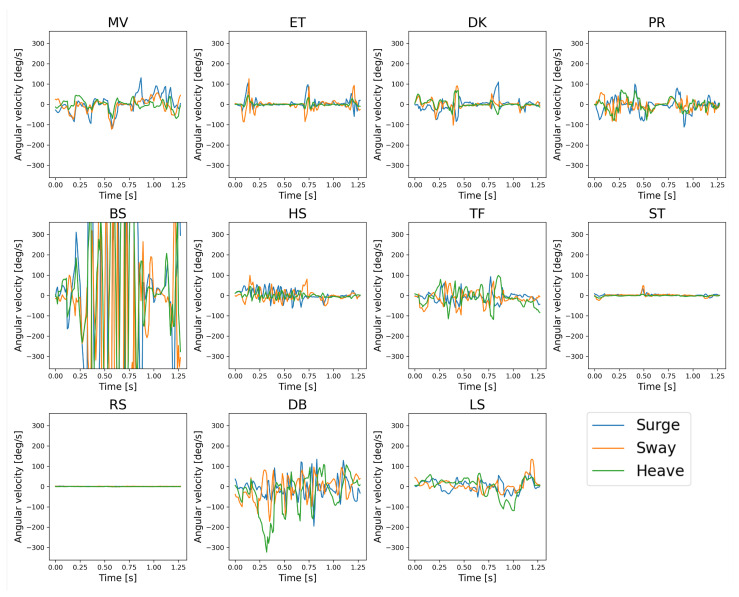
Examples of angular velocity signals of each behavior, down-sampled to 100 Hz.

Not all the collected data were labeled with the 12 behavior classes. A considerable bias exists in the probability of the occurrence of individual behaviors. Hence, frequent behaviors were partially sampled and labeled. Table 2 summarizes the collected data, including the number of labels (i.e., the observations of the target behaviors, the sum, the mean, and the standard deviation (SD) of the duration of the observations). The table exhibits that the mean duration varies from a few seconds to 30 s or more, depending on the behavior. This information was utilized to determine the window length in time in Section 3.2.2. The examples of collected acceleration and angular velocity signals are depicted in Figure 2 and Figure 3, respectively. The figures display that BS is quite the characteristic waveform. In addition, the resting behaviors, that is, ST and RS, appear to be distinguishable from other behaviors; however, the two classes might be easily mistaken. Furthermore, we discovered that the acceleration and angular velocity signals had correlated change patterns; however, the acceleration signal kept the posture information due to the gravitational information. The benefit of these sensors and the validity of the features calculated from the signals are subsequently verified.

## 3. Experiment

### 3.1. Evaluation Items

Behavior recognition follows a standard supervised machine learning process with training and run (test) phases. In the training phase, a particular classifier with the best hyperparameters via hyperparameter tuning learned the characteristics of the signal for each behavior. Then, the trained classifier model was utilized in the run phase. In both phases, the time-series data of raw acceleration and angular velocity signals are divided into fixed-size windows, and a multi-dimensional feature vector is calculated from each window. Note that our preliminary experiments with feature learning approaches (i.e., long short-term memory (LSTM) and convolutional LSTM [28]) showed relatively poor classification performance of 0.419 and 0.246, respectively. Thus, we took a feature engineering-based classification pipeline. We evaluated the following items in the experiment to investigate the impact on classification performance.

- Sampling frequency
- Windowing (the number of data points and the length (duration) of a window)
- Sensor modality
- Handling training data imbalance
- Classification model (classifier)
- Robustness against the difference in individual hens

### 3.2. Experimental System

**Figure 4:**
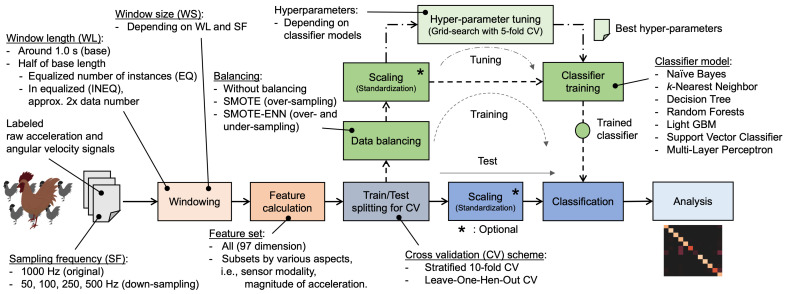
Processing flow. The underlined parts are evaluation items and their values.

Figure 4 illustrates the processing flow in the experimental system, where the underlined text represents the evaluation items described above and their values. The labeled dataset was used as input to the experimental system, and cross-validation (CV) schemes were applied to obtain reliable results for analysis. The following sections describe the design and implementation of the items; Python 3.10.4 running on Mac Pro (late 2013, CPU: Intel Xeon E5 3.5 GHz 6 Core, Memory: 32 GB, OS: macOS 11.6) implemented the experimental system.

#### 3.2.1. Sampling Frequency

The literature on behavior recognition of chicken utilizes a wide variety of sampling frequencies (SF) for body-mounted accelerometers (i.e., 5 Hz [24], 10 Hz [12], 20 Hz [13], 100 Hz [10,11,27], and 1000 Hz [26]). However, none of them demonstrated the impact of the frequency on the classification performance. In the experiment, we utilized a variety of SFs: 50 Hz, 100 Hz, 250 Hz, 500 Hz, and 1000 Hz. The frequencies except for 1000 Hz were down-sampled similarly as [29] (i.e., the data for 500 Hz, 250 Hz, 100 Hz, and 50 Hz were obtained by selecting the first sample in every 2, 4, 10, and 20 samples, respectively). The result is presented in Section 4.1.

#### 3.2.2. Windowing

As described in Section 2.3, the behavior start and end periods were recorded, and a particular label was assigned to the periods. Thus, the length of a behavior period is a factor for determining the window length (WL). Considering WL exceeds a labeled period, the window contains other behaviors that occur before and after the behavior, which may result in misclassification. Nonetheless, a short-length window generally degrades the classification accuracy [12,20,30]. Because the lower limit of the mean duration of behavior was approximately one second: 1.5, 1.3, and 1.4 s for DK, BS, and TF, respectively, as displayed in Table 2, we specify the base WL to “approximately” one second.

**Figure 5:**
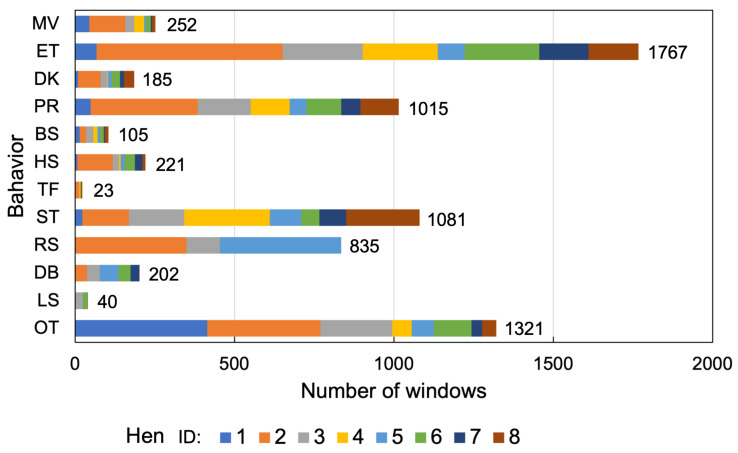
The distribution of the instances per behavior and individual hens.

To make a window fast Fourier transform (FFT)-friendly, the number of data points in a window (i.e., window size (WS)), should be a power of two. According to Equation (1), substantial WS and WL prepared for the evaluation were 64, 128, 256, 512, and 1024 samples and 1.28, 1.28, 1.024, 1.024, and 1.024 s for 50, 100, 250, 500, and 1000 Hz, respectively. A window was slid by 50% overlap between two consecutive windows. Because of the difference in the number of data points in a window due to SF, the number of instances for the classification differed by up to 25%. Therefore, the number of training and test data for each SF were standardized by down-sampling to correspond to the nominal one (at 50 Hz). Figure 5 summarizes the distribution of the number of instances per behavior and individual hens, which depicts the imbalance between classes and individuals.

(1) $$WL=\frac{WS}{SF}$$

In addition, *WL* was set to half of the base *WL* in the data of specific *SF* to evaluate the signal duration impact to be segmented as a window. This aspect resulted in the number of half-sized windows approximately doubling because of 50% overlapped sliding windowing. Therefore, to analyze the effect of *WL* and the number of instances in the dataset, classification in the halved windows was performed under two conditions: equalizing the number of instances to the case with base length windows (EQ) and without equalization (INEQ). We realized EQ by under-sampling the dataset of INEQ so that the number of instances in each behavior class could satisfy that of the base *WL*. Note that not doubling the number of data points (approximately 2 s) was to avoid further reducing the number of instances because some behaviors (BS, TF, and DK) had an average duration of fewer than 2 s, as listed in Table 2. The result is explained in Section 4.4.

#### 3.2.3. Classification Features

Classification features are crucial in determining a recognition system’s performance. The features convey behavior-specific information about chickens regarding posture, rotational movement, complexity, intensity, and swiftness of motion. We specified 14 features, as summarized in Table 3, often used in animal activity recognition [12,22,23,24,31,32,33,34] and human activity recognition [35,36,37,38].

We calculated the 14 features from the time and the frequency domains of acceleration and angular velocity signals to capture the characteristics of behaviors from different aspects. In addition to the three axes of an accelerometer (i.e., *x*, *y*, and *z*), we introduced the magnitude of the acceleration signal (the $l^{2}$-norm of a vector of three axes) as the fourth dimension (*m*) and an orientation-independent feature.

In total, 97 features were calculated, that is, (four axes from accelerometer and three axes from angular velocity sensor) × 13 types and (three combinations correlation of coefficients between two of three axes) × (accelerometer and angular velocity sensor). The effectiveness of the features in classification is evaluated per the following (sub) set, where the numbers in brackets represent the dimension of the features:

- Entire feature set (97)
- Acceleration-based features (55)
- Angular velocity-based features (42)
- Features without magnitude of acceleration (42)

The comparison between acceleration-based features and angular velocity-based features was expected to emphasize the significance of modality, which could result in the reduction of the sensor for energy conservation and miniaturization of the sensor node. Additionally, the significance of orientation-independent features was investigated by extracting the features derived from the magnitude of acceleration (i.e., the *m*-axis) from the entire dataset and comparing them with the result of the complete dataset. The result is explained in Section 4.5.

#### 3.2.4. Imbalance Data Handling

Because animals do not follow instructions as humans do in the data collection process for behavior recognition, the amounts of data in the behavior classes generally were excessively imbalanced. The classifiers were trained to reduce the overall error. Therefore, the trained models were biased in classifying the majority class satisfactorily. We applied training data sampling techniques to address this issue where the number of training data in each class is equalized (i.e., balanced, by either under-sampling or over-sampling, or both). We utilized synthetic minority over-sampling techniques (SMOTE) [39] so that the amount of training data of minority classes could be artificially increased by interpolating the nearest samples. In addition, we adopted a combination of SMOTE for over-sampling and edited nearest neighbors (ENN) for under-sampling, called SMOTEENN [40]. Here, ENN filters out majority class data as noise where only the data of the minority class exist in the neighborhood. We utilized imbalanced-learn 0.8.1 [41] to implement SMOTE and SMOTEENN. Note that the data balancing process was only applied to training data. The result is explained in Section 4.3.

#### 3.2.5. Classifier and Its Training

We applied classification models, which are often used for animal behavior recognition. The models use various learning concepts. We evaluated them using evaluation metrics to identify the most suitable algorithm. They include naïve Bayes (NB) as a probabilistic method, *k*-nearest neighbor (*k*NN) as an instance-based method, decision tree (DT) as a rule-based method, and random forests (RF) [42] as a bagging-based ensemble learning method. Additionally, light gradient boosting machine (LightGBM, in this study, simply LGBM) [43] was a boosting-based ensemble learning, support vector machines (SVM) [44] was a kernel function-based method, and multi-layer perceptron (MLP) was an artificial neural network-based method. Among them, RF demonstrated success in other studies of animal behavior recognition [15,22,27,45,46]. LGBM is a relatively new model, and very few examples in animal behavior recognition have used LGBM [24]; however, the effectiveness of LGBM in human activity recognition has been reported [47,48,49]. In MLP, one hidden layer was applied (i.e., a simple three-layered network).

Each classifier has inherent parameters that control the learning process, called hyperparameters. As described in Section 3.3.1, the classification performance was evaluated by cross-validation (CV) on the training and test data. To avoid overfitting the entire dataset, we adopt the nested CV approach [50,51]; the inner CV was applied to obtain the most suitable combination of hyperparameters, and the outer CV was used to evaluate the classifier model trained with the selected parameters. In this study, we performed the inner CV with a five-fold CV on the training data. The classifier models, including related processing (e.g., hyperparameter tuning and cross-validation) were implemented by scikit-learn 1.0.2 [52] and LightGBM 3.2.1 [53]. The hyperparameters and their candidate values are summarized in Table 4; the default values were used for other hyperparameters. Please refer to the dedicated API references for details of the hyperparameters, such as their meaning and default values.

As the scale of the features differs between features, we applied a standardization that extracts the mean and scale to unit variance; the parameters, that is, the mean and variance, were calculated from training data and used for training and test data. Scaling was not applied to tree-based classifiers (i.e., DT, RF, and LGBM).

Section 4.1 explains the classification performances using the seven classifiers with variable SFs.

### 3.3. Evaluation Schemes

#### 3.3.1. Cross Validation

As described in Section 3.2.5, we applied a nested CV where the outer CV is responsible for evaluating the classifier; the evaluation includes the classifier model and various aspects noted above, such as sampling frequency and window size. It is because the data used for training and testing rely on these elements. We performed two types of outer CV: 10-fold-CV and Leave-One-Hen-Out (LOHO)-CV. In 10-fold-CV, we randomly split the entire dataset into ten mutually exclusive subsets; we integrated nine subsets into one training dataset and used the remaining subset for testing the classifier. We iterated this process ten times by changing the test and training subsets combinations. The *k*fold-CV assumes that the distribution of features in the dataset is identical for the training and test data [54] and can be considered to represent the average performance of the classifier on the dataset. In actual use, it can also represent the performance under the condition that new data with similar feature distributions as the dataset are obtained, such as when data from the same population are newly provided for classification. Note that the entire dataset is split into ten subsets in a stratified method; therefore, each split contains almost similar proportions of classes as the original dataset.

LOHO-CV can examine the robustness against data distribution and individual differences. LOHO-CV was performed by testing a dataset from a particular individual using a classifier trained without a dataset from the individual. This process is iterated until all individuals become test individuals. Unlike *k*fold-CV, the training dataset does not contain data from a test individual. Thus, LOHO-CV was regarded as a more appropriate and practical evaluation method than *k*fold-CV because individual differences inherently exist among chickens. Particularly, the nested LOHO-CV approach is even more rigorous because the data from test individuals are never exhibited in the tuning and training process. Whereas in the non-nested LOHO-CV, the data leakage would occur when the hyperparameters are tuned using the data from test individuals. This leave-one-individual-out type of CV is also demonstrated in human activity recognition and is often called leave-one-subject-out (LOSO-CV) or leave-one-person-out CV (LOPO-CV).

We obtained a confusion matrix at each fold and calculated an average confusion matrix subsequently to the outer CV. A set of common performance metrics, described below, was then calculated from the confusion matrix.

#### 3.3.2. Performance Metrics

We evaluated the performance of the classification by three metrics: precision, recall, and F1-score. Precision refers to the degree to which the same result is obtained when repeated measurements were performed under similar conditions. Thus, precision can be considered an indication of the certainty of the judgment determined by the classifier. In contrast, recall indicates the degree to which the original class can be correctly detected. F1-score is a harmonic mean of the two metrics (i.e., precision and recall) to combine them into one and is often used as a metric for an imbalanced dataset. Thus, we utilized F1-score as a primary performance metric. Equations (2)–(4) represent precision, recall, and F1-score, respectively; $N_{correct_{k}}$, $N_{tested_{k}}$, and $N_{predicted_{k}}$ represent the number of cases correctly classified into $class_{k}$, the number of test cases in $class_{k}$, and the number of cases classified into $class_{k}$, respectively, whereas *k* is the index corresponding to 1 of 12 classes. For each of these three metrics, an average of 12 classes is calculated, which is called a macro-average metric (Equation (5)) and is unaffected by the majority classes because each class is considered equal weight.

(2) $$\begin{array}{ccc} precision_{k} & = & \frac{N_{correct_{k}}}{N_{predicted_{k}}} \end{array}$$

(3) $$\begin{array}{ccc} recall_{k} & = & \frac{N_{correct_{k}}}{N_{tested_{k}}} \end{array}$$

(4) $$\begin{array}{ccc} F1score_{k} & = & \frac{2}{1/precision_{k}+1/recall_{k}} \end{array}$$

(5) $$\begin{array}{ccc} macro-average\;metric & = & \frac{1}{12}\sum_{k=1}^{12}metric_{k} \end{array}$$

Furthermore, Equation (6) defines each metric individual independence ratio (IIR). IIR aims to evaluate the robustness of a model or behavior class to the training and test data consisting of different individuals. Smaller values of IIR indicate that classification performance generally is more individual-dependent. The metrics obtained by LOHO-CV also represent an individual’s robustness; however, we consider IIR that effectively compares the robustness among models and classes because of relative values.

(6) $$\begin{array}{ccc} IIR & = & \frac{metric\;by\;LOHO-CV}{metric\;by\;10fold-CV} \end{array}$$

To understand the impact of a particular condition, we define *a* difference of confusion matrices under two conditions by Equation (7), where *M*, *a*, and *b* represent the confusion matrix and conditions *a* and *b*, respectively. A positive value for a diagonal element indicates that condition *a* contributed more to the correct classification of the class than condition *b*. Moreover, a positive value for a non-diagonal element signifies that condition *a* increased misclassification. The visualization of $\Delta M_{a,b}$ in a heat map would allow us to understand the differences between conditions *a* and *b* in detail.

(7) $$\begin{array}{ccc} \Delta M_{a,b} & = & M_{a}-M_{b} \end{array}$$

As additional information for the classification system, we also measured the execution time for feature calculation and classification. In the experimental system, these processes were separated and performed as batch processing. Thus, the execution time was measured separately by obtaining the start and end timestamps of each process and dividing by the number of instances.

## 4. Results

### 4.1. Basic Classification Performance by Sampling Frequencies and Classifiers

Table 5, Table 6 and Table 7 list the F1-score, precision, and recall for 10-fold-CV and LOHO-CV schemes per sampling frequency (SF) and classifier, respectively. Regarding the result in 10-fold-CV, LGBM demonstrated the highest F1-score and precision at 1000 Hz and on average, whereas MLP was highest in recall at 250 Hz. However, LOHO-CV had the highest F1-score and recall with MLP and precision with LGBM at 100 Hz. LGBM, SVM, and MLP were the top three for both CV methods regarding F1-score and recall for the mean values per classifier. Moreover, for precision, RF, LGBM, and MLP were the top three for 10-fold-CV; RF, LGBM, and SVM were the top three for LOHO-CV.

The trends of the best and worst SF varied by classifiers. Considering the F1-scores of the top three classifiers, that is, LGBM, SVM, and MLP, the averages for each SF were as follows, beginning from 50 Hz: 0.868, 0.883, 0.879, 0.877, and 0.880 for 10-fold-CV and 0.663, 0.694, 0.686, 0.657, and 0.659 for LOHO-CV; the highest values were obtained at 100 Hz for both evaluation methods.

IIRs of the F1-score by SF and models are presented in Table 8, where all models had the highest IIR at 100 Hz or 250 Hz, and MLP exhibited the highest IIR of 0.804 at 100 Hz. Furthermore, the highest average IIRs per model and SF were obtained by MLP (0.779 and 0.747) at 250 Hz, respectively. However, MLP had the highest average IIR for each SF (0.786) for the classifiers with the top three highest average IIRs, that is, LGBM, SVC, and MLP. The subsequent evaluation is based on the performance of these three classifiers at 100 Hz. In particular, the results obtained by MLP at 100 Hz sampling data are regarded as the baseline because of the highest IIR. Section 4.2 presents a detailed performance of the baseline.

The processing time for feature calculation is presented in Table 9 and varied by SF because the number of data points in a window varied (i.e., 64, 128, 256, 512, and 1024 for 50 Hz, 100 Hz, 250 Hz, 500 Hz, and 1000 Hz, respectively); the feature calculation time increased linearly with SF. Table 10 shows the classification time per classification model. DT was the fastest, followed by MLP, NB, LGBM, RF, SVM, and kNN; however, when the time for feature calculation and classification were combined, more than 99% of the time was spent on feature calculation. Thus, we can say that there is no difference between classifiers in execution time.

### 4.2. Detail Classification Performance in Baseline Case

**Figure 6:**
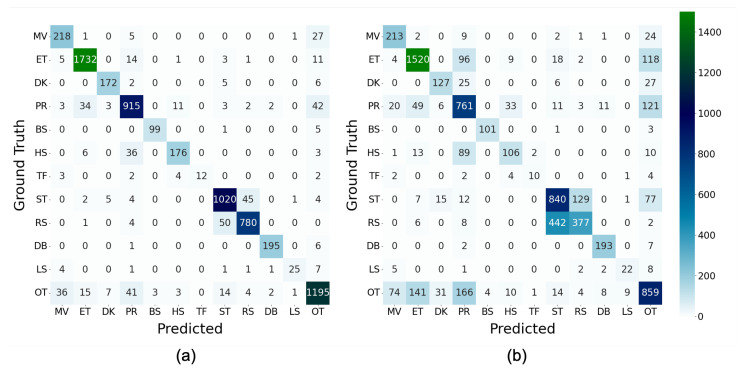
Confusion matrices in the baseline condition (MLP, 100Hz, 128 samples): (**a**) 10-fold-CV and (**b**) LOHO-CV.

Figure 6 depicts the confusion matrices of the baseline, where mutual confusion between ET and PR, PR and HS, ST and RS, MV and OT, ET and OT, and PR and OT was discovered in 10-fold-CV; considerable confusion existed in LOHO-CV. The common characteristics in these confusion matrices are that the heads of the hen moved, which could generate identical movement patterns in acceleration and angular velocity. Additionally, because behaviors such as pecking sensors and other individuals were included in the OT, confusion between ET, PR, and OT may have occurred.

**Figure 7:**
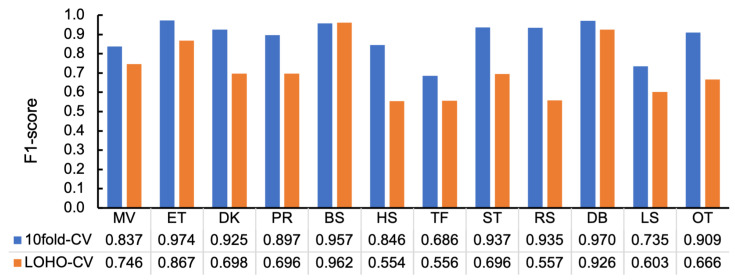
F1-scores per behavior class in the baseline condition (MLP, 100Hz, 128 samples).

Figure 7 depicts the F1-scores per behavior class calculated from these confusion matrices, which show that the F1-scores of 10-fold-CV ranged from 0.686 for TF to 0.974 for ET, whereas those of LOHO-CV ranged from 0.554 for HS to 0.926 for DB, indicating that the values varied from behavior to behavior. The Pearson’s correlation coefficient between the values of 10-fold-CV and LOHO-CV is 0.634. This indicates similar trends between the two evaluation schemes. The difference between the values of 10-fold-CV and LOHO-CV is considered to represent the robustness to the differences in training and test data; the smaller the value, the more robust it is. In Figure 7, there are some cases, such as BS and DB, with almost no difference, whereas there are other cases with large differences, such as HS, ST, RS, and OT.

### 4.3. Classification Performance by Imbalance Data Handling Methods

**Figure 8:**
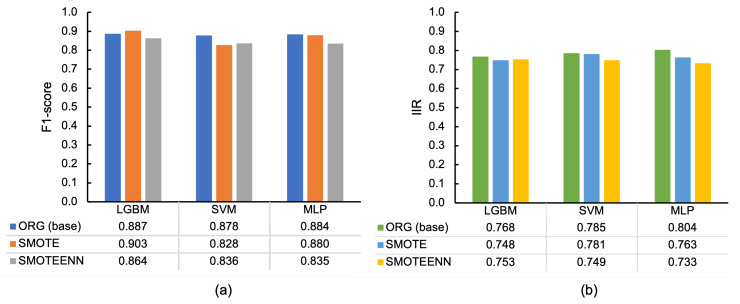
Classification results by imbalance handling techniques with different classifiers (128 samples at 100 Hz): (**a**) F1-scores in 10-fold-CV and (**b**) IIRs of F1-scores.

Figure 8a,b depict F1-scores and IIRs of F1-scores by imbalance data handling techniques per classifiers, where ORG represents the original condition and equals the case with the rows at 100 Hz in Table 5 and Table 8 for 10-fold-CV and IIR, respectively. In Figure 8a, it illustrates that the highest values were obtained in ORG (i.e., original imbalance data) in SVM and MLP, followed by SMOTE except for SVM. In contrast, in Figure 8b, the ORG condition achieved higher values than SMOTE and SMOTEENN. This outcome signifies that the data without balancing are less affected by individual differences.

**Figure 9:**
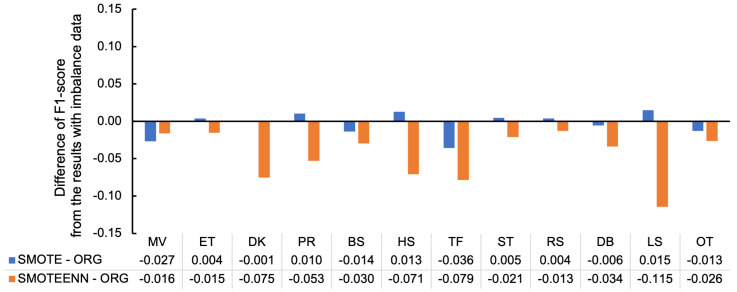
Difference of F1-scores between imbalance handling techniques and ORG conditions per behavior classes with 10-fold-CV in baseline condition.

Figure 9 emphasizes the effects of imbalance handling by subtracting the F1-score of each behavior class in the ORG condition from that of SMOTE or SMOTEENN, where the positive values indicate that the techniques successfully increased the F1-score. Considering the figure, SMOTE improved the F1-scores of 6 out of 12 classes. However, no improvement in the F1-score was discovered in SMOTEENN.

**Figure 10:**
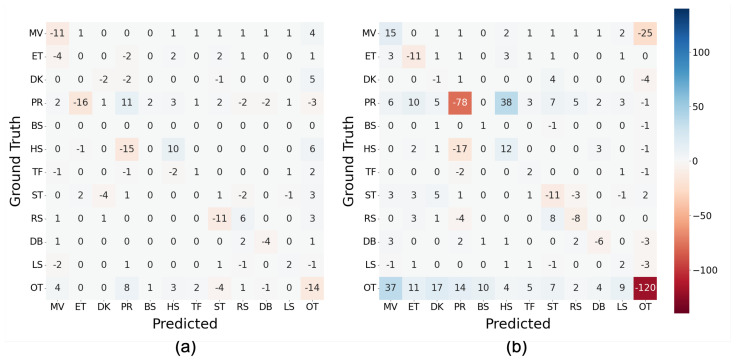
Differences between confusion matrices of classification with balancing and without balancing (ORG) (MLP, 128 samples at 100 Hz): (**a**) $\Delta M_{SMOTE,ORG}$ and (**b**) $\Delta M_{SMOTEENN,ORG}$.

Figure 10 depicts the difference in confusion matrices between SMOTE and ORG ($\Delta M_{SMOTE,ORG}$) (Figure 10a) and between SMOTEENN and ORG ($\Delta M_{SMOTEENN,ORG}$) (Figure 10b). Based on the definition of ΔM, SMOTE (Figure 10a) successfully improved the classification between PR and ET, PR and HS, and ST and RS, observed in Figure 9. In contrast, the correct classification of MV was decreased by SMOTE primarily due to the increase in mutual misclassification with OT. The confusion with OT was often discovered between other classes by locating positive values in the row and column of OT. Regarding SMOTEENN (Figure 10b), correct classification into MV, BS, HS, TF, and LS was increased, whereas that of ET, PR, ST, and OT decreased; the misclassification of PR, HS, ST, RS, and OT into other classes increased.

### 4.4. Classification Performance by the Window Length and Number of Training Instances

**Figure 11:**
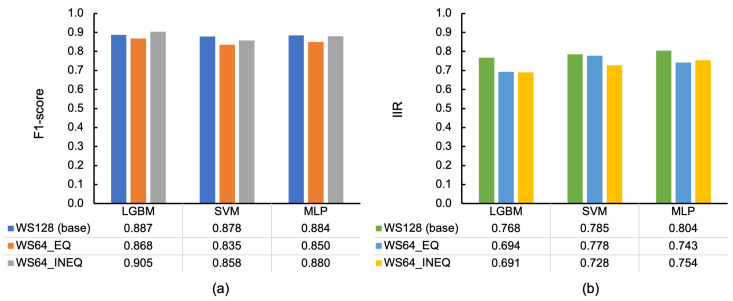
Classification results by WS (128 and 64 samples at 100 Hz) and the equality of the number of data to the baseline (EQ for equal and INEQ for inequal conditions): (**a**) F1-scores in 10-fold-CV and (**b**) IIRs of F1-score.

WS was halved into 64 (sampled at 100 Hz), indicating that the classification features were calculated in a WL of 0.64 s. Figure 11a depicts the F1-scores of the base condition (WS128), halved WL with an equal number of instances to the base condition (WS64_EQ), and halved WL with an inequal (double) number of instances to the base condition (WS64_INEQ) per classifier by 10-fold-CV, where the highest F1-scores were obtained in WS128, except for INEQ of LGBM, and the lowest values were obtained in WS65_EQ in all cases. IIRs are illustrated in Figure 11b, where the base condition demonstrated the highest values in all classifiers, whereas the second highest relied on the models: WS64_EQ in LGBM and SVM and WS64_INEQ in MLP, with very minimal differences except for SVM (0.05). This aspect indicates that WS, and accordingly WL, was more significant than the number of instances for the characteristics of training data.

**Figure 12:**
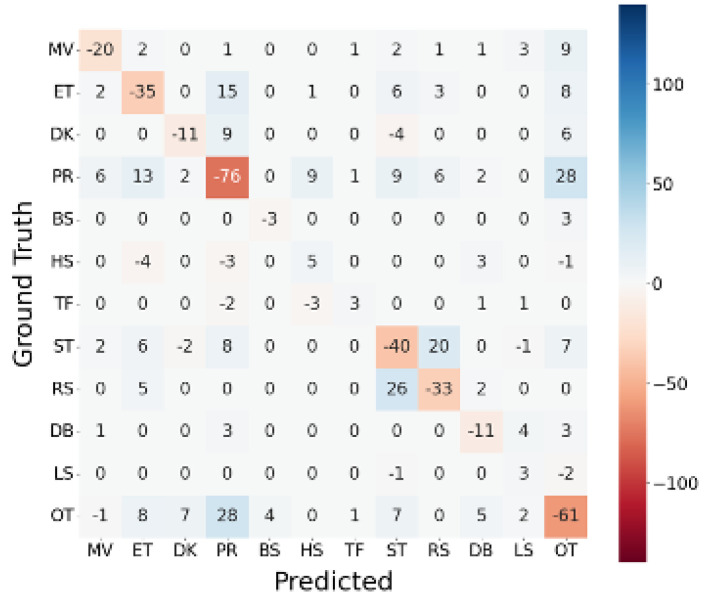
Differences between confusion matrices of WS64_EQ and WS128: $\Delta M_{WS64\_EQ,WS128}$ (MLP at 100 Hz).

Furthermore, the difference between the confusion matrices of WS64_EQ and WS128, $\Delta M_{WS64\_EQ,WS128}$, is depicted in Figure 12. Mutual confusion between PR and ET, PR and ST, PR and OT, and ST and RS increased in WS64_EQ. These pairs indicate that classification might be more challenging with a window of 0.64 s than 1.28 s. In contrast, HS, TF, and LS were more successfully classified in WS64_EQ than in WS128, signifying that these classes are less likely to be negatively affected even with short windows.

### 4.5. Effective Feature Group

**Figure 13:**
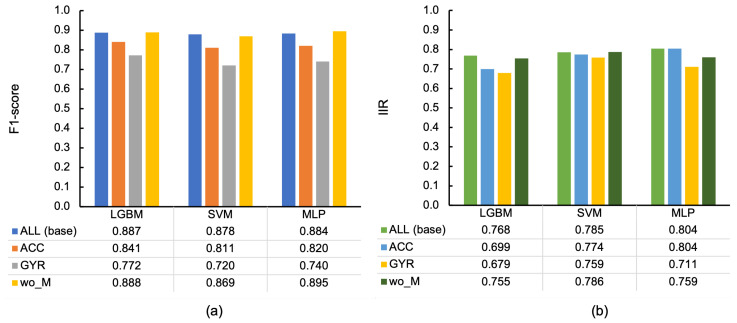
Classification results by by feature set with different classifiers, sampled at 100 Hz: (**a**) F1-scores in 10-fold-CV and (**b**) IIRs of F1-score. ALL: all features, ACC: acceleration-derived features, GYR: angular velocity-derived features, wo_M: features eliminating magnitude of three-axis acceleration.

Figure 13 depicts the F1-scores in 10-fold-CV (Figure 13a) and IIRs of the F1-score (Figure 13b) for each feature set with different classifiers. ACC and GYR comprise particular sensor modalities of acceleration and angular velocity, respectively, to understand the contribution of these modalities in classification. However, the feature set wo_M was generated by extracting the features calculated using the magnitude of three-axis acceleration from the entire feature set (ALL). The comparison between ALL and wo_M was expected to explore the significance of the orientation-independent amount of force.

Our analysis demonstrated that the features derived from acceleration alone were insufficient, although they contributed more to the classification than the features derived from angular velocity in all classifiers. In addition, the magnitude of acceleration did not contribute to LGBM and MLP in 10-fold-CV due to higher F1-scores than those of ALL; however, the lower IIRs in LGBM and MLP indicate a significance of the magnitude of acceleration in individual-independent classification. The heterogeneity of the feature space due to the increase in dimensionality may have contributed to the absorption of individual differences rather than overfitting.

**Figure 14:**
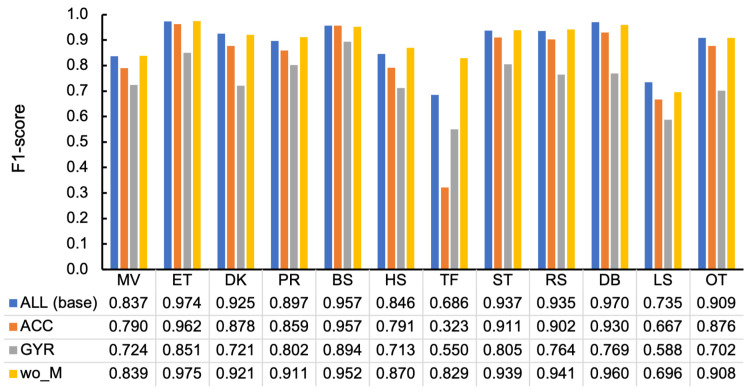
F1-scores per behavior class by feature set in MLP with 10-fold-CV, sampled at 100 Hz. ALL: all features, ACC: acceleration-derived features, GYR: angular velocity-derived features, wo_M: features eliminating magnitude of three-axis acceleration.

**Figure 15:**
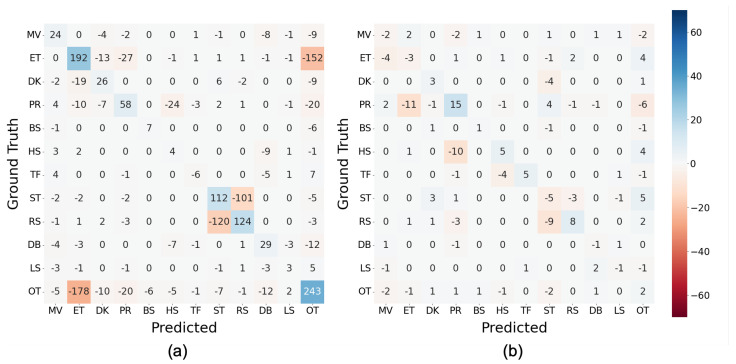
Differences between confusion matrices of classification (**a**) with features derived from acceleration and angular velocity ($\Delta M_{ACC,GYR}$) and (**b**) without magnitude of acceleration and with all features ($\Delta M_{wo\_M,ALL}$) (MLP at 100 Hz).

Figure 14 depicts the breakdown per behavior class in MLP. Overall, using all the acceleration and angular velocity features resulted in higher scores than using them separately. Regarding the acceleration and angular velocity features comparison, all cases in ACC except for TF exhibited higher values than GYR. Figure 15a illustrates the difference between confusion matrices of the classification using ACC and GYR (i.e., $\Delta M_{ACC,GYR}$). The matrix represents how the differences of F1-scores per behavior class were expedited in more detail; the mutual confusion between ET, DK, PR, and OT was decreased in ACC, in addition to another mutual confusion between ST and RS.

In addition, the increase in wo_M compared to ALL was discovered in 7 out of 12 behavior classes in Figure 14. In particular, TF was considerably larger than that of ALL by 0.143, which may be why wo_M in MLP had a higher average score than that of ALL. Figure 15b depicts the difference between confusion matrices of the classification using wo_M and ALL (i.e., $\Delta M_{wo\_M,ALL}$). Regarding TF noted above, the number of misclassifications into HS was decreased by four, and that of correct classification increased by five, resulting in considerable improvement of the F1-score of TF because the number of TF instances was inherently minimal (40), as illustrated in Figure 5.

## 5. Discussion

### 5.1. Classifiers

We sampled seven supervised classifiers often used in human and animal behavior recognition. As presented in Section 4.1, LGBM, SVM, and MLP demonstrated generally high classification performance, although there were some differences depending on the performance metrics, SF, and evaluation schemes. Recent literature reveals the superiority of LGBM [24,48,49], and LGBM demonstrated the highest F1-score at 1000 Hz (0.898) and the average in all classifiers (0.887). However, in LOHO-CV, the average score degraded to 0.660 and exhibited the lowest score of the three classifiers. This outcome resulted in the IIRs of LGBM being the smallest of all SFs of the three models.

The decrease in classification performance in LOHO-CV indicates the difference in feature distribution between training and test data resulting from individual hens’ differences. The difference in the experiment resulted from the difference in the classifier because the training and test data were unified for all classifiers in a particular SF. As described in Section 3.3.1, the hyperparameters of the classifiers were adjusted in the process of nested CV to avoid data leakage from the test data. We utilized the data sampled from the training data to evaluate a particular set of hyperparameters, and thus the classifier was fit with data composed of different populations of hens than the test data. We consider LGBM to be the most over-trained of the three classifiers, whereas MLP was the least likely to be over-trained. The 10fold CV approach assumes that the distribution of training and test data is identical. Therefore, it is applicable when the study is conducted on a specific population and training and testing are limited to that population. Otherwise, it is applicable if the training data can be collected from a sufficiently diverse population to include data from individuals with a distribution similar to that of the unknown individuals tested. In these cases, we conclude that LGBM is worth using.

### 5.2. Sampling Frequency

Section 4.1 explained the sampling frequency for the highest F1-scores in the top three classifiers obtained at 100 Hz for 10-fold-CV and LOHO-CV. An average IIR of the top three models also exhibited the highest value of 0.786 at 100 Hz, subsequently 0.780 and 0.764 at 250 and 50 Hz, respectively. Higher sampling frequencies can improve the temporal and spatial resolution of the signal and are considered to increase the expressiveness of the signal features for each action. However, noise and individual differences may be accentuated. It may be because the IIR for the top three classifiers increased at 100 Hz or 250 Hz and decreased at 500 Hz and 1000 Hz, specifically, the large decrease from 0.768 to 0.722 for LGBM. However, further investigation is required to determine the exact reasons for this.

### 5.3. Handling Imbalance Data

As demonstrated in Section 4.3, the balancing techniques did not exhibit an overall improvement in F1-score compared with the original imbalance data except for LGBM. The analysis of F1-scores in 10-fold-CV of the baseline condition (Figure 9) shows that SMOTE improved classification performance in half of the classes; however, the degradation of MV, BS, TF, and OT outweighed the improvement. Although these classes, except for OT, were minority classes, the number of instances was increased to match that of ET by approximately 7, 17, and 77, respectively. We consider that the increase did not result in clarification of the decision boundaries between classes.

### 5.4. Window Size (Length) and the Number of Training Instances

Section 4.4 explained the trend of the F1-score regarding WS (WL) and the number of instances. First, it indicates that increasing the number of training instances by decreasing WS contributed to the classification performance in 10-fold-CV (WS64_INEQ > WS64_EQ). It is reasonable because numerous training data generally facilitate learning the characteristics of the data. The F1-score of WS64_INEQ of LGBM was highest in the three classifiers; however, its IIR was lowest, implying an effect of overfitting of LGBM, as noted in Section 5.1. Second, the patterns of WS128 outperforming WS64_EQ in all situations and WS128 outperforming WS64_INEQ in SVM and MLP suggest that a window of 1.28 s may convey more information than a window of 0.64 s, although a halved window could generate about twice as many training examples. However, it is challenging to generalize that 1.28 s is the most satisfactory WL for the hen’s behavior recognition because we have not tested other combinations of WL and the number of instances.

The depiction of $\Delta M_{WS64\_EQ,WS128}$ in Figure 12 highlights the impact of halving the windows on classification in more detail. As shown in Figure 2 and Figure 3, the reasons for the increase in mutual confusion between ET and PR in WS64_EQ may be as follows. Several bursts (three for ET and two for PR) are discovered in the figures; however, decreasing the WL decreased the number of bursts in a window and is presumed to result in difficulty distinguishing between behaviors. In ET and PR of the figures, only one burst exists in the first half of the 0.64 s window. Head movements in ST, where the hens stand still but occasionally look around, could result in the sensor noticing movements similar to preening. This aspect would result in difficulty distinguishing between the two behaviors in a short window, as was the case for ET. However, several short windows created in ST that contained only a stationary state without bursts in Figure 2 and Figure 3 might increase the confusion with RS, which is a stationary state with almost no head movement. In addition, the increase in mutual confusion between many behaviors and OT might be because the shorter window had more patterns similar to OT, primarily composed of various behaviors. Moreover, HS and TF had similar or adequate classification accuracy in a shorter window. The reason for this is unclear; however, it appears to be related to the longer burst duration. These discoveries imply that window size has a large impact on hens’ behavior classification, and decreasing the window size can increase mutual confusion between different behaviors.

### 5.5. Effective Sensor Modalities and Importance of Magnitude of Acceleration

Section 4.5 explains that, regardless of the classifier, GYR had lower F1-scores than ACC in the 10-fold-CV due to increased mutual confusion between ET, DK, PR, and OT and between ST and RS in GYR compared to ACC. The increase in confusion between ET and OT in GYR may be because OT includes the action of pecking sensors of other individuals. Although feeding and pecking are similar because they produce short impulse-like bursts, the direction of head movement differs between feeding in the bait box and pecking at another individual’s sensor due to the different positions of the target. An angular velocity sensor measures the relative change in angle, whereas the force applied to the sensor, including the acceleration of gravity, is obtained by an accelerometer, and an accelerometer could therefore discriminate these differences in the direction of movement. Similarly, the increased confusion in the GYR between ET, DK, and PR may have resulted from their inability to discriminate between different target locations for beak use but a commonality in the act of beak use.

As for ST and RS, ST is primarily a stationary behavior compared to the other behaviors, although hens occasionally move their heads when standing. In RS, the body may tilt when lying on the ground, resulting in a variation in sensor posture compared to standing firmly on both legs. Therefore, we believe an accelerometer is more effective than an angular velocity sensor because an accelerometer can capture postural information from the gravitational acceleration component and the force associated with the movement. Moreover, TF was the only behavior with a higher value in GYR than in ACC. An angular velocity sensor may have been more effective because tail wagging was mostly in a horizontal plane. Thus, although an accelerometer demonstrated a high overall classification performance, the combination with the angular velocity sensor (i.e., ALL) resulted in classification that is more accurate. Although an angular velocity sensor generally has a higher power consumption than an accelerometer [55], we suggest the combination of the gyro sensor with an accelerometer if there is no battery limitation.

Similar to the above, in Section 4.5, more than half of the comparisons between wo_M and ALL in Figure 14 depict that wo_M had higher F1-scores. Particularly, in TF with a value of 0.143 higher, it might be the primary reason why the macro average of wo_M was higher than that of ALL. The detail of classification in TF is depicted in Figure 6a with four, three, two, and two misclassifications out of 23 instances of TF for HS, MV, PR, and OT, respectively. In addition, they were reduced by four, zero, one, and one by excluding the magnitude of acceleration. Moreover, the number of misclassifications to LS increased by one; however, the number of correct classifications into TF increased by five. HS is a rapid head-scratching behavior with one foot; therefore, the force applied to the body might be similar to that of TF, even if the direction of movement was different from that of TF that wags the tail at high speed. Therefore, we believe that the misclassification was suppressed by extracting the magnitude value and emphasizing the directional component; however, further experiments with a larger sample are required to determine the misclassification source because the number of instances is only 23. Figure 15b exhibits that the number of correct classifications into LS decreased by one, whereas the number of misclassifications to LS increased by one in MV, TF, and DB and decreased by one in ST. This resulted in a 0.039 lower F1-score for LS, compared to ALL, as depicted in Figure 14; however, it is challenging to generalize the cause of this difference because the individual differences were only plus or minus one.

### 5.6. Existing Work on Chicken Behavior Recognition

We discuss the results compared with other work related to chicken behavior recognition. Table 11 summarizes relevant parameters discovered in each work: the number and type of behaviors, sensor modality, number of axes, sampling frequency, window length, classification method, and condition of data.

The present study addressed 11 types of behaviors belonging to categories such as migration, feeding, self-defense, grooming, resting, and searching and is characterized by its aims to quantify various daily behaviors of chickens compared to other studies. In the works of Abdoli et al. [10,11] and Quwaider et al. [13], a class PK was among the classification targets we did not address. However, because Abdoli et al. used the term “pecking” along with “feeding” in their study, it can be noted that our method also covered what they called pecking. In Quwaider et al.’s study, the target of PK appears to be the ground, sensors, etc. In the data collected in this study, we also discovered some behaviors of pecking at other individuals’ sensors as strange objects; however, we included these behaviors in OT. Note that the introduction of OT was unique to our study. In a classification task in machine learning, the input data are classified into one of the learned classes. Thus, miscellaneous data obtained in the automatic processing of long-term data, expected in practical applications, would be incorrectly classified into a behavior class with similar characteristics. In this study, the behaviors included in OT contain pecking at the sensor and various movements such as balance breaking, beak scratching, and looking around, as listed in Table 1. We believe the introduction of OT would improve the discrimination accuracy of the 11 types of behaviors other than OT. However, there are still movements that are not included in OT. Therefore, the reliability of the classification results would be further improved by post-processing the classifier output, such as using the rejection option. The research group of Derakhshani and Shahbazi classified various behaviors into three classes based on their intensity [26,27], which was intended to be used to control the amount of fine dust produced by hens’ activity or to assess the health and welfare status of hens. We focused on the recognition of individual behaviors because we intend to use the frequency and transitions of the behaviors and the locations where the behaviors occur in our attempts to improve hen’s welfare.

Regarding the sensor modality, all existing work except Li et al. [24], Shahbazi et al. [26], and ours used only an accelerometer for behavior classification. Li et al. performed feature selection by integrating acceleration and angular velocity-derived features and did not investigate the classification performance for each sensor. However, we generated a subset of features for each modality (ACC and GYR) and compared the performance with the combined set (ALL). Our results demonstrated that the accelerometer is more beneficial than the angular velocity sensor as a single sensor and that the combination of the two is excellent. The results showed an overall trend and a trend for each action. This outcome provides information to decide whether to adopt or reject the angular velocity sensor, considering the power constraints of the measurement system and the type of behavior to emphasize. Shahbazi et al. further combined a magnetic field sensor and confirmed that it was more accurate than using the combination of accelerometer and angular velocity sensor in an artificially generated high-level noise environment. We consider it an interesting finding, and the applicability is worth considering while taking into account the influence of metals present in the actual rearing environment on the magnetic field sensor.

For sampling frequency, we down-sampled data collected at 1000 Hz to generate data sampled at five different frequencies for comparison, whereas other studies used a single value. Although the optimal frequency varied with the classifier, we concluded that 100 Hz was suitable from an individual-independent perspective. The results showed a trend in classification accuracy at frequencies lower and higher than this value, which will be beneficial in determining sampling frequency in future research.

Similar comparisons were made for window length; Banerjee et al. [12], Yang et al. [25], and Derakhshani et al. [27] also compared two, six, and two different window lengths, showing 4 s, 1 s, and 4 s as better window length than the other candidates, respectively. For datasets collected in finite time, decreasing the window length increases the number of data instances, which increases the training data. Therefore, the number of instances required standardization to investigate the effect of the window length. Contrary to other studies, we compared a window of 1.28 s and 0.64 s with and without standardizing the number of instances based on these ideas. We demonstrated that 1.28 s is more effective regarding informativeness even when the number of training instances is approximately doubled in 0.64 s. We did not compare the results over 3 s in these studies because we utilized an upper limit of about 1 s based on the duration of the target behaviors. Therefore, it is challenging to generalize the results. Nevertheless, we could show a lower bound of about 1 s.

We compared traditional classifiers, such as *k*NN and NB, and newer ones, such as LGBM, and discovered that LGBM, SVM, and MLP were superior regarding F1-scores in 10-fold-CV and IIR. The 10-fold-CV approach assumes identical data distribution during training and testing, and IIR acts as an indicator of the independence of individual differences. The superiority of these classifiers was also found to be beneficial by Banerjee et al. [12], Yang et al. [25], and Li et al [24]. In the literature above, only [26] utilizes a deep learning (DL)-based approach (i.e., convoluational neural networks (CNNs)). As described in Section 3.1, we did not take DL-based approaches due to relatively poor classification performance in a preliminary evaluation, which we consider because of the small number of data. We are currently developing an interactive labeling tool using the present behavior recognition pipeline to accelerate making a large dataset. The evaluation with DL-based approaches remains for future work.

To address the imbalance in the training data, we compared the classification performance using training data balanced by over-sampling and training data that remained imbalanced. The results demonstrated that the training with imbalanced data was satisfactory. Collecting labeled data in animal behavior recognition is more challenging than in human behavior recognition because cooperation from the target (i.e., animals) is more demanding to obtain, and imbalance is more likely to occur [10]. We consider the comparison significant: it demonstrated that balancing training data did not necessarily positively influence classification performance. However, data balancing should be implemented per new datasets because the result depended on data distribution.

**Figure 16:**
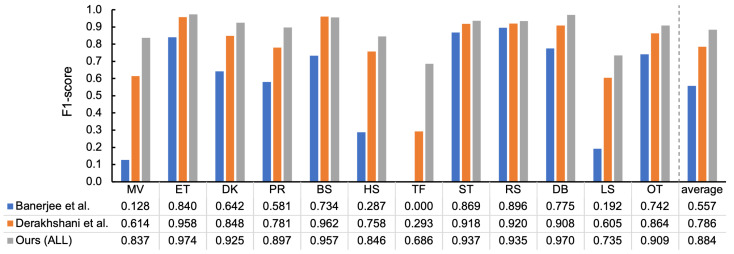
The result of applying the features used in existing work to our classification pipeline (MLP at 100 Hz, 10-fold-CV).

To understand the applicability of features used in existing work to our recognition task and dataset, the features used in the work of Banerjee et al. [12] and Derakhshani et al. [27] were evaluated. The reasons for the choice of these studies are that they took a feature engineering-based approach using fixed-size windows and they had sufficient information for reproduction. In the work of Banerjee et al. [12], ENTR and MEAN were calculated from each of the *x* and *y* axes of an accelerometer; these four features were included in our feature set. A total of 31 features calculated from the acceleration signal were taken from Derakhshani et al.’s work: SKEW, KURT, MEAN, SD, variance (VAR), MIN, MAX, ENTR, ENGY, and covariance (COV) for each of three axes and the average signal magnitude (ASM), in which all but VAR, COV, and ASM were a subset of our feature set. Figure 16 shows the F1-scores per behavior, in which the result of our features with the highest score (i.e., ALL) are also presented as a comparison. Compared to the results of Derakhshani et al., our feature set (ALL) showed higher F1 scores for all behaviors but BS, including the mean, particularly MV, TF, and LS, and the scores of their ET, ST, RS, and OT were comparable to ALL. On the other hand, most scores using Banerjee et al.’s features were considerably lower than ALL, but comparable for stable states such as ST and RS.

To summarize, we increased the number of target behaviors, compared different parameters that should be considered when designing a hen behavior recognition system using wearable sensors, and demonstrated the superiority or inferiority of the candidate parameters. We also showed that the sensor modality and features used in existing work were insufficient to successfully classify the target behaviors. Considering this, we assume it would contribute beneficial information for designing similar systems.

### 5.7. General Discussion

The generality of the results of this study to hen behavior recognition is discussed. In this study, we collected data from Boris Brown layers. However, we believe the results apply to other breeds of layers and roosters, as there are almost no differences in their behavioral patterns. However, there may be differences depending on the age in weeks. When using the same acceleration sensor device, it may be challenging to distinguish the behavior of a newborn hen because the sensor is large and heavy for its body size. In addition, its movements are limited and different from the learned patterns; however, we consider that there will be no problem when the hens are approximately three months old or later when they begin to lay eggs.

In addition, although we collected data in an area of 100 cm × 76 cm, we assume our results are applicable even if the hens are kept in a larger space because their basic behaviors, such as dust bathing and feeding, do not change. Because the hens’ movements are expected to be more dynamic in a larger space due to their long strides, it may be possible to detect them accurately by retraining the classifier through data collection. Similarly, the extra space will enable the hens to jump upwards; thus, we expect to notice movements that could not be collected. As noted above, we believe that we can redirect data from multiple behaviors; therefore, additional data collection limited to new behaviors will allow us to include many behaviors in the recognition target.

## 6. Conclusions

We investigated a method of realizing a behavior recognition system for continuous monitoring of hen’s behavior over a long period, which aimed at studying a hen-rearing system based on animal welfare. We specified 12 behavior classes of 11 types of daily and miscellaneous activities and developed a classifier using supervised machine learning. Several significant components of the processing pipeline from the measured raw data to the final behavior class were considered as parameters, including the classifier, sampling frequency, window length, data imbalance handling, and feature group and sensor modality. We specified a reference configuration based on the result: an MLP classifier using features derived from an accelerometer and an angular velocity sensor calculated in a 1.28 s window at 100 Hz sampling without balancing the training data. This configuration has the greatest potential to achieve highly accurate hen behavior recognition in unbalanced environments with large individual differences, such as the present dataset.

This study targeted more behaviors and verified the effect of more parameters than previous studies in chicken behavior recognition using wearable sensors. However, not all parameter combinations were exhaustively explored during the study, and validation was conducted with fixed classifiers, sampling frequencies, window lengths, etc. Therefore, the truly optimal parameter combinations were unidentified. Nevertheless, we consider that the results are significant and can be utilized to recognize certain behaviors, design similar systems, and estimate the impact of particular parameters on the system’s performance. As an application of this work, we are currently working on a method to evaluate rearing systems to improve hens’ welfare by visualizing and quantifying the frequency of behaviors and the patterns of transition among behaviors.

## Figures and Tables

**Table 1:** Behavior types and their descriptions.

Behavior	Symbol	Category	Description
Moving	MV	Migration	Walking or running to another place
Eating	ET	Intake	Eating feed by own beak from feeder
Drinking	DK	Intake	Drinking water from water cup
Preening	PR	Self-defence	Arranging or oiling own feather by own beak
Body shaking	BS	Grooming	Shaking body with standing
Head scratching	HS	Grooming	Scratching own head by one leg with standing
Tail flapping	TF	Grooming	Flapping tail right and left with standing
Stopping	ST	Resting	Stopping with standing
Resting	RS	Resting	Lying down and being still
Dust-bathing	DB	Grooming	Head-rubbing and scratching litter by leg
			with lying down
Litter scratching	LS	Seeking	Scratching litter by leg on floor with standing
Others	OT	N/A	Other behaviors such as pecking sensor, stretching,
			breaking balance, beak scratching, head shaking,
			jumping up and down perches and nest boxes
			looking around, etc.

**Table 2:** Summary of collected data.

	MV	ET	DK	PR	BS	HS	TF	ST	RS	DB	LS	OT
Number of labeled duration	95	78	183	264	105	156	23	29	12	134	37	650
Sum of duration [s]	433.4	2321.5	280.9	1669.0	141.2	425.7	31.7	1470.2	1071.9	468.7	80.3	2157.7
Mean duration [s]	4.6	29.8	1.5	6.3	1.3	2.7	1.4	50.7	89.3	3.5	2.2	3.3
SD of duration [s]	3.0	28.0	0.4	7.2	0.3	1.3	0.3	48.8	95.1	1.0	0.8	13.9

**Table 3:** Classification features.

Name	Symbol	Description
Minimum [22,23,24,32,33,35]	MIN	Minimum value of time-series data
Maximum [22,23,24,32,33,35]	MAX	Maximum value of time-series data
Mean [12,22,23,24,31,32,33,35,36,37]	MEAN	Average of time-series data
Standard deviation [22,23,24,31,32,33,35,36,37]	SD	Standard deviation of time-series data
Skewness [32,33]	SKEW	Skewness of time-series data that represents the degree of asymmetry
		of the signal distribution
Kurtosis [22,23,24,31,32,33]	KURT	Kurotis of time-series data that represents the degree of peakedness
		of the signal distribution
Inter-quartile range [22,23,24,31,32,35]	IQR	Difference between the third quartile and the first quartile in time-series data
Mean absolute deviation [32]	MAD	Average of absolute difference from mean in time-series data
Median absolute deviation [35]	MedAD	Median of absolute difference from median in time-series data
Mean crossing [18,24,36]	MCRS	The number of crossing the mean value
Correlation coefficient [35,36,37]	CORR	Pearson’s correlation coefficient of signals from two axes in time-series data
Spectral energy [33,35,37]	ENGY	Sum of the squared discrete component in energy spectrum
Frequency entropy [12,22,23,33,35,37]	ENTR	Frequency entropy
Dominant frequency [22,24]	DOMIF	Frequency that gives maximum magnitude in the frequency spectrum

**Table 4:** Hyperparameters and their candidate values in each classifier model.

Classifier Model	Class Names in Scikit-Learn	Hyperparameters	Candidate Values
Naïve Bayes (NB)	GaussianNB	N/A	N/A
k-Nearest Neighbor (kNN)	NearestNeighbors	n_neighbors	1, 2, 3, 4, 5, 6, 7, 8, 9, 10
		weights	uniform, distance
Decision Tree (DT)	DecisionTreeClassifier	max_depth	5, 7, 10, 12, 15, 20
		criterion	gini, entropy
		spitter	best, random
RandomForest (RF)	RandomForestClassifier	n_estimators	100, 200, 300
		criterion	gini, entropy
		max_depth	5, 10, 50
		max_features	sqrt, log2
LightGBM (LGBM)	LGBMClassifier	min_child_samples	0, 5, 15, 300
		num_leaves	15, 31, 127
		reg_alpha	0, 0.1, 1.0, 10.0
		reg_lambda	0, 0.1, 1.0, 10.0
Support Vector Machines (SVM)	SVC	C	0.01, 0.1, 1.0, 10.0
		kernel	linear
Multi-layer Perceptron (MLP)	MLPClassifier	hidden_layer_sizes	50, 75, 100
		learning_rate_init	0.0001, 0.001, 0.01
		alpha	0.00001, 0.0001, 0.001
		early_stopping	True

**Table 5:** F1-scores per sampling frequencies (SF) for different evaluation schemes and classifiers. Text in bold style indicates the highest scores in each column. Furthermore, the underlined text indicates the highest value in each CV scheme.

SF [Hz]	10-fold-CV							LOHO-CV						
	NB	*k*NN	DT	RF	LGBM	SVM	MLP	NB	*k*NN	DT	RF	LGBM	SVM	MLP
50	**0.599**	0.854	0.741	0.841	0.866	0.862	0.877	0.406	0.612	0.506	0.587	0.651	0.674	0.665
100	**0.599**	0.863	**0.776**	0.854	0.887	**0.878**	**0.884**	0.400	0.638	0.514	0.597	**0.681**	**0.690**	**0.710**
250	0.581	0.866	0.748	0.849	0.889	0.869	0.879	0.403	0.**643**	0.529	0.636	0.673	0.687	0.698
500	0.590	0.859	0.765	0.870	0.896	0.874	0.862	0.389	0.616	0.515	0.631	0.645	0.651	0.675
1000	0.594	**0.872**	0.760	**0.872**	**0.898**	0.871	0.872	**0.408**	0.620	**0.535**	**0.651**	0.648	0.668	0.660
Mean	0.593	0.863	0.758	0.857	0.887	0.871	0.875	0.401	0.626	0.520	0.620	0.660	0.674	0.682

**Table 6:** Precision per sampling frequencies (SF) for different evaluation schemes and classifiers. Text in bold style indicates the highest scores in each column. Furthermore, the underlined text indicates the highest value in each CV scheme.

SF [Hz]	10-fold-CV							LOHO-CV						
	NB	*k*NN	DT	RF	LGBM	SVM	MLP	NB	*k*NN	DT	RF	LGBM	SVM	MLP
50	**0.582**	0.885	0.747	0.920	0.912	0.891	0.908	0.433	0.650	0.511	0.628	0.711	0.726	0.698
100	0.581	**0.906**	**0.773**	0.927	0.923	**0.902**	**0.926**	0.427	0.650	0.524	0.709	**0.755**	**0.743**	**0.741**
250	0.572	0.894	0.756	0.922	0.937	0.894	0.881	0.437	0.670	0.536	0.737	0.732	0.719	0.724
500	0.575	0.886	0.771	0.923	0.934	0.894	0.889	0.417	0.630	0.517	0.745	0.707	0.687	0.695
1000	0.578	0.893	0.760	**0.930**	**0.940**	0.894	0.878	**0.441**	**0.672**	**0.546**	**0.751**	0.718	0.709	0.690
Mean	0.578	0.893	0.761	0.924	0.929	0.895	0.896	0.431	0.654	0.527	0.714	0.725	0.717	0.710

**Table 7:** Recalls per sampling frequencies (SF) for different evaluation schemes and classifiers. Text in bold style indicates the highest scores in each column. Furthermore, the underlined text indicates the highest value in each CV scheme.

SF [Hz]	10-fold-CV							LOHO-CV						
	NB	*k*NN	DT	RF	LGBM	SVM	MLP	NB	*k*NN	DT	RF	LGBM	SVM	MLP
50	0.720	0.840	0.737	0.818	0.839	0.843	0.855	0.473	0.612	0.521	0.589	0.636	0.660	0.660
100	**0.722**	0.842	**0.780**	0.825	0.864	**0.861**	0.859	0.469	**0.640**	0.524	0.596	0.659	0.675	**0.700**
250	0.707	0.851	0.743	0.828	0.860	0.850	**0.877**	0.476	**0.640**	0.537	0.624	**0.660**	**0.682**	0.689
500	0.714	0.844	0.760	0.843	**0.871**	0.859	0.844	0.468	0.615	0.525	0.624	0.642	0.648	0.671
1000	0.710	**0.858**	0.760	**0.846**	**0.871**	0.855	0.867	**0.479**	0.611	**0.538**	**0.637**	0.642	0.661	0.655
Mean	0.715	0.847	0.756	0.832	0.861	0.854	0.860	0.473	0.623	0.529	0.614	0.648	0.665	0.675

**Table 8:** IIRs by SF with different classifiers. Text in bold style indicates the highest scores in each column; underlined text indicates the highest value of all.

SF [Hz]	NB	NN	DT	RF	LGBM	SVC	MLP	Mean (All)	Mean (Top-3)
50	0.677	0.717	0.682	0.697	0.752	0.781	0.758	0.724	0.764
100	0.668	0.740	0.662	0.698	**0.768**	0.785	**0.804**	0.732	**0.786**
250	**0.693**	**0.742**	**0.707**	**0.749**	0.757	**0.790**	0.794	**0.747**	0.780
500	0.660	0.717	0.673	0.725	0.720	0.745	0.783	0.718	0.751
1000	0.687	0.711	0.704	0.746	0.722	0.766	0.758	0.728	0.757
mean	0.677	0.725	0.686	0.723	0.744	0.774	0.779	0.730	0.768

**Table 9:** Time for feature calculation by SF.

SF [Hz]	50	100	250	500	1000
Calculation time [$\times 10^{-3}$ s/instance]	21.446	24.369	29.237	40.180	58.660

**Table 10:** Time for classification per model.

Model	NB	*k*NN	DT	RF	LGBM	SVM	MLP
Classification time [$\times 10^{-3}$ s/instance]	0.007	0.196	0.001	0.082	0.027	0.112	0.002

**Table 11:** Comparison with work on behavior recognition using wearable sensors. Underlines indicate the values reported as having the best classification performance on average.

Literature	Behavior ^1^ (Number and Type)	Sensor ^2^	SF [Hz]	WL [s]	Classification ^3^	Data ^4^
[13]	6 (DB, PK, PR, RS, ST, MV)	A (2)	20	1	thresholding	N/A
[12]	6 (ET, DB, DK, RS, ST, MV)	A (2)	10	3, 4	MLP, RBF, DT, NB	IMB
[10]	3 (ET/PK, DB, PR)	A (3)	100	variable	NN	IMB
[11]	3 (PK, DB, PR)	A (3)	100	variable (DB, PR),	NN with	IMB
				1 (PK)	thresholding	
[25]	4 (ET, DK, MV, RS)	A (3)	40	1, 3, 5, 7, 10, 20	kNN, SVM	B
[24]	2 (ET, DK)	A (3), G (3)	5	2.8	LR, XGBoost,	IMB
					DT, NB, LGBM	
[27]	3 (L, M, H)	A (3)	100	1, 4	RF, BT, s*k*NN	IMB
[26]	3 (L, M, H)	A (3), G(3), M(3)	1000	0.025, 0.05, 0.1	CNNs	IMB
Our work	12 (MV, ET, DK, PR, BS, HS,	A (3), G (3)	50, 100, 250	0.64, 1.024, 1.28	kNN, NB, DT, RF,	B, IMB
	TF, ST, RS, DB, LS, OT)		500, 1000		LGBM, SVM, MLP	

^1^ PK: Pecking. L: Low-intensity, M: Moderate-intensity, H: High-intensity behaviors. Other abbreviations follow those in Table 1. ^2^ A: Accelerometer, G: Gyro sensor (angular velocity sensor), M: Magnetic field sensor. The numbers in parentheses indicate the number of axes. ^3^ CNN: Convolutional Neural Network, BT: Bagged Trees, s*k*NN: subspace *k*NN. Other abbreviations follow those in Table 4. ^4^ Condition of training data. IMB: Imbalanced, B: Balanced.

## Data Availability

The data presented in this study are available from the corresponding author on reasonable request.

## Abbreviations

The following abbreviations are used in this manuscript:

BT	Bagged Trees
CNN	Convolutionl Neural Network
CV	Cross-validation
DT	Decision Tree
ENN	Edit Nearest Neighbors
LightGBM	Light Gradient Boosting Machine
LOHO-CV	Leave-One-Hen-Out Cross-Validation
LR	Logistic Regression
LSTM	Long Short-Term Memory
MLP	Multi-Layer Perceptron
NB	Naïve Bayes
NN	Nearest Neighbor
RBF	Radial Basis Function
RF	Random Forests
SD	Standard Deviation
SMOTE	Synthetic Minority Over-Sampling Techniques
SVM	Support Vector Machines
WL	Window Length in Time
WS	Window Size (Number of Data Points)
XGBoost	eXtreme Gradient Boosting
